# Exploring the Experiences and Reproductive Health Needs of Young Women With Premature Ovarian Insufficiency: A Qualitative Content Analysis

**DOI:** 10.1002/hsr2.71705

**Published:** 2026-01-11

**Authors:** Farideh Pargar, Maryam Farjamfar, Shahrbanoo Goli, Zeinab Rabiei, Zahra Motaghi

**Affiliations:** ^1^ Student Research Committee, School of Nursing and Midwifery Shahroud University of Medical Sciences Shahroud Iran; ^2^ Clinical Research Development Unit, Imam Hossein Hospital Shahroud University of Medical Sciences Shahroud Iran; ^3^ Center for Health Related Social and Behavioral Sciences Research Shahroud University of Medical Sciences Shahroud Iran; ^4^ Department of Midwifery, School of Nursing and Midwifery Bushehr University of Medical Sciences Bushehr Iran; ^5^ Reproductive Health Department, School of Nursing and Midwifery Shahroud University of Medical Sciences Shahroud Iran

**Keywords:** content analysis, premature ovarian insufficiency, qualitative study, reproductive health

## Abstract

**Background:**

Premature ovarian insufficiency (POI) is defined as the loss of ovarian activity before age 40, resulting in hypoestrogenism, hypergonadotropic hypogonadism, amenorrhea, and infertility, significantly impacting women's reproductive and emotional health. This study explores the experiences and reproductive health needs of women with POI in Iran.

**Methods:**

A qualitative study using conventional content analysis was conducted. Data were collected through semi‐structured, in‐depth interviews with 20 participants (14 women with POI, 3 specialists, and 3 healthcare providers) from infertility and health care centers in Tehran and Ahvaz, Iran, via purposeful sampling until data saturation. The participants were informed that the interview will be recorded, transcribed verbatim in Persian, and analyzed using MAXQDA 2024 software.

**Results:**

Six themes emerged from the data analysis: Psychological Distress and Identity Loss, Compromised Physical and Systemic Health, Deprivation of Reproductive Autonomy, The Experience of Premature Aging, Barriers to Integrated Healthcare, and Lack of Comprehensive Social Support.

**Conclusion:**

The findings of this study reveal that the experience of Iranian women with premature ovarian insufficiency extends beyond purely medical dimensions, encompassing deep psychological, social, and legal challenges. These women grapple with feelings such as psychological failure stemming from the loss of fertility and the sensation of aging prematurely. Furthermore, the analysis highlights their “Deprivation of Reproductive Autonomy” and a critical need for comprehensive supportive systems that are not currently accessible, a finding tied directly to the themes of “Barriers to Integrated Healthcare” and “Lack of Comprehensive Social Support.”

## 1 Introduction

Premature ovarian insufficiency (POI) is characterized by the loss of ovarian activity before age 40, leading to hypoestrogenism, hypergonadotropic hypogonadism, amenorrhea, and infertility [1, 2]. Affecting approximately 3.7% of women under the age of 40 globally, with variations observed across different ethnic groups, POI has profound physical and psychological consequences [3]. Beyond the physiological consequences, POI has a significant psychosocial impact, affecting women's quality of life, body image, and sense of identity due to the loss of fertility [4]. The early cessation of ovarian function and the resulting hypoestrogenism elevate the risk of several long‐term health complications. These include an increased risk of cardiovascular disease, osteoporosis, cognitive decline, diabetes, cancer, and premature mortality, with the severity of these risks linked to the age of onset [5, 6]. Leen Be Gold study has shown that POI has numerous short and long‐term complications and consequences, such as sexual dysfunction, reproductive health, and Systemic Health [7].

In addition to the significant physical health burden, the psychological and emotional impacts are extensive. The link between stigma perception and depressive symptoms, which is well‐documented in the broader psychiatric literature, is particularly relevant here. Women with POI often experience anxiety, depression, reduced resilience, and significant marital discord, all of which are frequently exacerbated by the pervasive social stigma associated with infertility [8, 9]. The estrogen deficiency also causes sexual dysfunction, including vaginal dryness, loss of elasticity, dyspareunia, and avoidance of sexual activity, further complicating personal and relationship well‐being [10, 11].

Hormone replacement therapy (HRT) is considered the cornerstone of POI management [12]. This is strongly emphasized by evidence‐based guidelines from leading international organizations, including the American Society for Reproductive Medicine (ASRM, 2020) [13], European Society for Human Reproduction and Embryology (ESHRE, 2016, last edited 2023) [14], and the British Menopause Society (BMS) [15].

While HRT effectively alleviates hypoestrogenism symptoms and mitigates long‐term health risks, its implementation in many regions, including Iran, remains limited. This is primarily due to a combination of cultural stigma, a lack of awareness among both patients and healthcare providers, and resource constraints within the healthcare system [16].

Despite the significant impact of POI, comprehensive healthcare resources for its management remain largely inadequate, and there has been minimal progress in understanding the full spectrum of its consequences, particularly in specific cultural contexts [5]. Continued research is needed to optimize their long‐term health and quality of life with timely intervention [14, 16]. Therefore, a deeper understanding of the lived experiences of these women is essential to develop more effective and culturally sensitive interventions. The ultimate goal is to identify the basic needs and uncover the holistic dimensions of reproductive health for women with POI to inform better care.

## 2 Materials and Methods

### 2.1 Study Design

A qualitative study employing a conventional content analysis approach was conducted to explore the lived experiences and reproductive health needs of women with POI. This inductive methodology allowed categories and themes to emerge directly from the data, remaining grounded in the participants' perspectives without a pre‐existing theoretical framework [17]. The research was carried out at infertility and healthcare centers across two major Iranian cities, Tehran and Ahvaz, to capture a diverse range of experiences from different socioeconomic and cultural contexts.

### 2.2 Participants

Participants were purposively selected from these centers and included women with POI, specialists, and Healthcare professionals. The diagnostic criteria for POI, confirmed by a gynecologist, required at least 4 months of amenorrhea and elevated serum FSH levels (more than 40 international units per milliliter) from two separate samplings taken 1 month apart. Exclusion criteria for women with POI included chronic debilitating diseases or diagnosed mental illnesses. Specialists and healthcare providers with at least 3 years of experience were also included in the study. Sampling continued until data saturation, resulting in a total of 20 participants. The sample composition is detailed in.

### 2.3 Data Collection

Semi‐structured, in‐depth interviews were conducted in Persian from June to December 2024, lasting 30–60 min. Interviews were held privately at infertility centers or via telephone due to POI's low prevalence. The first author (F.P.) conducted all interviews, which were recorded and transcribed verbatim after obtaining informed consent.

### 2.4 Data Analysis

Data were analyzed using an inductive conventional content analysis approach with MAXQDA 2024 software. This method was chosen to allow themes and categories to emerge directly from the data without relying on a predefined theoretical framework. The process began with the first author (F.P.) conducting initial coding.

To ensure the dependability and confirmability of the findings, the initial codes were reviewed by two experienced qualitative researchers (SH.G. and Z.R.). The research team held regular meetings to discuss and refine the codes, deliberating on any discrepancies until a consensus was reached. This process of investigator triangulation minimized individual bias and enhanced the objectivity of the findings.

The trustworthiness of the study was further established using Lincoln and Guba's criteria [18]. Credibility was achieved through prolonged engagement with the data and member‐checking, where initial findings were shared with a few participants to ensure they resonated with their experiences. To ensure transferability, a thick description of the study's context, participants, and cultural setting was provided, allowing other researchers to assess the applicability of the findings. Dependability was ensured by maintaining a detailed audit trail that documented the entire research process, from data collection to final analysis. Finally, confirmability was maintained by clearly distinguishing between participants' voices (through direct quotes) and the researchers' interpretations, with peer review helping to minimize researcher bias.

Saturation was determined by two key indicators. First, thematic redundancy, where data collection and analysis continued until no new themes or sub‐themes emerged from the latest interviews. Second, we used to code stability, considering saturation achieved when our main codes stabilized and new data could be easily categorized into the existing framework. We also assessed saturation separately for each subgroup to ensure we had comprehensively captured their distinct perspectives.

### 2.5 Ethical Considerations

This study, part of a PhD thesis, was authorized by Shahroud University of Medical Sciences. The study protocol was reviewed and approved by the Ethics Committee of the university (IR.SHMU.REC.1400.015). Prior to each interview, we explained the research objectives to the participants, and informed consent was obtained from all individuals. Participants were informed that the interviews would be audio‐recorded but that they had the right to stop the recording at any point. They were also assured that their personal information would be kept strictly confidential.

To ensure the confidentiality and anonymity of participants, a consistent coding system was used for the quotations. Accordingly, participants with POI were designated with codes P1 to P14, specialists with codes S1 to S3, and healthcare providers with codes H1 to H3. All direct quotations in the results section are cited using these identifiers.

## 3 Results

Findings were derived from a data analysis of face‐to‐face and telephone interviews conducted with 14 women (9 married, 5 unmarried), 3 healthcare providers, and 3 specialists. The participants' ages ranged from 28 to 52, and the time since their diagnosis with POI was between 1 and 12 years. Demographic characteristics are presented in Tables 1 and 2. Six themes emerged from the data analysis, which are detailed in Table 3.

**Table 1:** Summary of participant characteristics (*N* = 14).

Variables	Details
Age range	At Assessment: 26–39; At Interview: 28–52
Education Level	< 12 years: 4; Bachelor's: 8; Master's: 2; PhD/Physician: 2
Position	Housekeeper: 3; Employed: 11
Causes	Idiopathic: 10; Autoimmune: 1; Surgery: 1; Chemotherapy: 1; Pharmacological: 1
Hormonal treatment	Received: 4; Not Received: 7; Unable to Take: 3
Marital status	Married: 9; Unmarried: 5
Number of children	No children: 8 participants; One child: 3 participants; ≥ 2 children: 3 participants

**Table 2:** Summary of specialists and healthcare providers.

Participant ID	Role	Specialty	Experience (Years)
S1	Specialist	Infertility specialist	> 10
S2	Specialist	Gynecologist	> 15
S3	Specialist	Nutritionist	> 5
H1	Healthcare provider	Midwife	> 7
H2	Healthcare provider	Midwife	> 5
H3	Healthcare provider	Midwife	> 8

**Table 3:** Summary of experiences and reproductive health needs.

Category	Main category (Theme)	Subcategory	Key codes	Representative quotation
Experiences	1. Psychological distress and identity loss	Mood fluctuations	Anger, irritability, insomnia, loneliness	“Most days I am angry. I become very irritable and aggressive at work…”
		Feelings of ineffectiveness	Feeling like a burden, job loss, wasting time and money	“I feel like a low status in front of my husband. I can neither be a good wife nor a mother…”
		Conflicts in maternal feelings and infertility	Desire to have children, longing to experience pregnancy	“The fact that I am infertile eats me from the inside like a termite…”
	2. Compromised physical and systemic health	General unhealthy	Osteoporosis, joint pain, forgetfulness, weight gain…	“My doctor said an MRI has shown that one of my lumbar vertebrae has osteoporosis…”
		Infertility	Irregular menses, shrinking ovaries, sudden diagnosis…	“I was found infertile before I thought about getting married and suddenly was confronted with a severe limitation.”
	3. Deprivation of reproductive autonomy	Reproductive choice	Concerns about marital stability, divorce, inability to choose sex of child…	“We decided to have a planned pregnancy… But we were confronted with a big shock.”
		Sexual well‐being	Sexual dissatisfaction, painful intercourse, vaginal dryness…	“I am infertile, so I do not deserve to have pleasant sex. The relationship for me is very annoying…”
	4. The experience of premature aging	Subjective age	Loss of youth and vitality, feeling of crumbling from the inside…	“I am crumbling inside.”
		Ambiguous future	Gray life, lack of goals and inspiration, loneliness…	“I have dreams, but my life is gray. I am lonely…”
Reproductive health needs	5. Barriers to integrated healthcare	Diagnosis	Confusion, vague diagnosis, repeated tests…	“I went to different doctors, but they all spoke vaguely. None of them could tell me clearly what had happened…”
		Evaluation	Lack of regular check‐ups, absence of guidelines…	“The doctor told me that if you are infertile, take supplements and do an annual checkup. That's it.”
		Comprehensive management	Referral to different specialists, feeling of being lost…	“Every day I was referred to a doctor… I got tired.”
		Family planning	Lack of awareness of alternative options, concerns about egg donation…	“I am afraid of adoption and egg donation. I might not feel like a mother enough.”
	6. Lack of comprehensive social support.	Registry system	Unawareness of the problem's magnitude, “unknown” patients…	“We don't have an exact cognition of the magnitude of this problem.”
	Perceived sensitivity	Patients' lack of awareness, insufficient information among providers…		“I've recently realized that lot of my problems are related.”

### 3.1 Psychological Distress and Identity Loss

Most Participants reported psychological distress, which included negative self‐concept and emotional turmoil. This theme was supported by the following subcategories:

1. Mood fluctuations: Participants frequently experienced a range of emotions, including shock, denial, anger, and depression. As a 38‐year‐old unmarried woman with idiopathic POI explained, “I'm angry most days and jealous of married people, but sometimes feel quiet and helpless” (P7).
2. Feelings of ineffectiveness: Many women felt inadequate in their roles as wives and potential mothers. One participant with idiopathic POI captured this sentiment, stating, “My dream of being a good mother is dead” (P10).
3. Conflicts in maternal feelings and infertility: Participants expressed a profound conflict between their desire to experience motherhood and the reality of their condition. For example, one participant with a bicornuate uterus noted, “I want to experience pregnancy, but my bicornuate uterus makes it impossible” (P14, POI diagnosis was idiopathic). In general, A bicornuate uterus generally does not preclude pregnancy; however, it is associated with an elevated risk of miscarriage and preterm delivery.
4. Loss of concentration: Some women reported a significant decline in their ability to focus. One participant with endometriosis and POI described this struggle, saying, “I'm restless and can't focus. It's part of my personality now” (P4).

### 3.2 Compromised Physical and Systemic Health

Most Participants reported a range of physical complications, including general health deterioration, menopause‐related issues, and infertility.

1. General health deterioration: Participants reported various health issues such as osteoporosis, weight gain, and cognitive decline. A participant with lupus described her experience, stating, “My MRI showed osteoporosis. I have severe pain and vaginal atrophy” (P3).
2. Menopause complications: Many women experienced symptoms of estrogen deficiency, including vaginal dryness and signs of premature aging.
3. Infertility: For some, the infertility diagnosis presented a significant life challenge. As one woman with lupus and POI stated, “I was found infertile before marriage, a severe limitation” (P3).

### 3.3 Deprivation of Reproductive Autonomy

Participants faced significant challenges to their reproductive health, impacting marital stability and sexual well‐being.

1. Reproductive autonomy and marital stability: Infertility often led to marital strain and emotional distress. One woman shared, “My husband threatens to take a second wife, and my family agrees. I feel trapped” (P6, idiopathic). Another expressed, “I fear my husband will divorce me because I can't have children” (P9, idiopathi
2. Sexual well‐being: Estrogen deficiency frequently caused sexual dysfunction. A participant noted, “My vagina is dry, and sex is painful” (P8, idiopathic). However, some women, such as P1, P5, and P6, reported minimal impact on their sexual well‐being, showing resilience.

### 3.4 The Experience of Premature Aging

Most participants felt they had aged prematurely. This experience was supported by the following subcategories:

1. Subjective age: Many women described feeling physically and emotionally older than their chronological age. As one participant with idiopathic POI expressed, “I'm crumbling inside” (P1).
2. Ambiguous future: The diagnosis of POI created a sense of an uncertain future, marked by both anxiety and hope. One participant highlighted her concerns about future motherhood, stating, “Even if I get pregnant, my baby wants a young mother” (P11, idiopathic). Despite these challenges, some women also expressed a sense of resilience. For example, one participant noted a blend of disappointment and hope, saying, “I'm disappointed but still hopeful for treatment” (P14, idiopathic).

### 3.5 Barriers to Integrated Healthcare

Participants highlighted several critical issues related to their diagnosis and care.

1. Diagnosis and evaluation: Many participants experienced significant delays in receiving a clear diagnosis. As one woman with idiopathic POI noted, “The doctors spoke vaguely. I was going crazy” (P8). This was compounded by a lack of adequate follow‐up care. Another participant with idiopathic POI reported being told simply to “take supplements and do checkups” (P12).
2. Comprehensive management: Fragmented and uncoordinated care was a major concern for participants. One woman with idiopathic POI described her frustration, stating, “I was referred to different doctors daily” (P1).
3. Family planning: Cultural resistance to options like adoption or oocyte donation was a noted challenge. A specialist described this sentiment, stating: “We can't safely stop taking birth control pills.” (S1, S2).

It should be noted that this specific statement reflects the individual perspective of a specialist and does not represent a global medical consensus. According to current international guidelines, such as those from ESHRE (2016, updated 2023), HRT is recommended as the primary approach for managing POI due to its greater clinical benefits and lower risks. However, combined oral contraceptives (COCs) may be considered a reasonable alternative in cases where contraception is required or to enhance patient adherence.

### 3.6 Lack of Comprehensive Social Support

Need for a Registry System: A major barrier to effective management of POI in Iran is the absence of a dedicated registry system for this condition. As one specialist noted,”We do not fully understand the scale of the problem” (S1, S3, H3).

This lack of data in the Iranian context hinders policymakers from accurately assessing the needs of this population. In contrast, some countries, such as those in the Nordic region, have established national registry systems to support diagnosis and manage drug reimbursement rights.

1. Need for a registry system: A major barrier to effective management was the absence of a dedicated registry system for POI. As one specialist noted, “We don't fully understand the problem's scale” (S1, S3, H3). This lack of data prevents policymakers from accurately assessing the needs of this population.
2. Perceived lack of sensitive care: Providers themselves expressed a sense of inadequacy in their ability to offer comprehensive care beyond standard medical prescriptions. One provider stated, “We don't do much except prescribe supplements” (H1, H2, S2). This sentiment underscores the need for better training and a more holistic approach to care.

## 4 Discussion

The rising prevalence of POI globally necessitates targeted interventions [3, 13, 16]. This study identified six themes reflecting the experiences and needs of women with POI in Iran.

### 4.1 Psychological Distress and Identity Loss

Participants' psychological distress, including anxiety and depression, aligns with prior research [19, 20, 21]. However, our findings suggest that unlike some studies reporting lower depression in women with a greater sense of purpose [22], the cultural expectations of motherhood in Iran intensified this emotional turmoil. This indicates that the social context plays a crucial role in shaping the psychological experiences of women with POI.

Physical complications, such as osteoporosis and cardiovascular risks, are consistent with the known effects of POI [5, 10, 17, 23, 24]. Our findings highlight the low prevalence of HRT use among Iranian women with POI, with only a limited number of participants (4 out of 14) receiving this treatment. This is likely attributable to cultural barriers, limited awareness, and restricted access to healthcare services. Meanwhile, international guidelines, such as those from the American Society for Reproductive Medicine (ASRM, 2020) and the European Society for Human Reproduction and Embryology (ESHRE, 2016, updated 2023), underscore the importance of HRT for managing symptoms and mitigating long‐term complications of POI [13, 14].

### 4.2 Deprivation of Reproductive Autonomy

Infertility and sexual dysfunction, amplified by cultural stigma, align with prior findings in other contexts [25, 26]. However, our study highlights challenges associated with cultural barriers for women with POI. In particular, unmarried women in our sample faced unaddressed issues related to sexual function, reflecting significant cultural constraints on open discussions about female sexuality [27, 28].

### 4.3 The Experience of Premature Aging

Participants' sense of premature aging aligns with studies reporting feelings of hopelessness in women with similar conditions [25, 29]. However, our findings also revealed instances of resilience, suggesting a potential for supportive interventions to mitigate these negative perceptions.

The cultural expectations of motherhood and the stigma surrounding infertility in Iran significantly contributed to these negative perceptions. This was further exacerbated by limited access to psychological support and comprehensive healthcare, leading to heightened feelings of hopelessness, a finding that contrasts with settings offering more robust support systems [25, 26]. For instance, our participants' sense of isolation was intensified by societal pressures, unlike in contexts where women have better access to counseling and support groups [8, 30]. Therefore, culturally tailored interventions, including patient education and support groups, are urgently needed to address these unique challenges.

### 4.4 Lack of Comprehensive Social Support

Evidence‐based guidelines from organizations like ESHRE (2016, updated 2023), ASRM (2020), and BMS emphasize HRT as a cornerstone of POI management. HRT is crucial for reducing symptoms and mitigating long‐term risks such as cardiovascular disease and osteoporosis [13, 14, 15]. However, our findings revealed that limited awareness and access to HRT in Iran, driven by cultural stigma and resource constraints, were evident in the participants’ experiences [27].

Furthermore, the fragmented care reported by our participants underscores the critical need for a multidisciplinary, patient‐centered approach to POI management [30, 31, 32, 33]. These gaps in care could be effectively addressed through the establishment of comprehensive health centers and the implementation of standardized protocols.

### 4.5 Lack of Comprehensive Social Support

National registry systems in individual countries are critical for accurately estimating the global prevalence of POI and enhancing its management.

management [3, 16]. Several participants and providers noted a perceived inadequacy of data and a lack of awareness surrounding the condition, with one specialist stating, “We lack sufficient knowledge of the problem's true scale.” This lack of understanding is compounded by the fact that many women with POI do not seek out care and treatment services. Furthermore, supportive communication from families and providers is critical to help women cope with the psychological and emotional burden of POI [30]. This statement emphasizes the necessity for enhanced education of healthcare professionals on international principles for the treatment of POI, such as those outlined by the European Society for Human Reproduction and Embryology (ESHRE, 2016, updated 2023) and the American Society for Reproductive Medicine (ASRM, 2020), to ensure a more holistic and evidence‐based approach to care [2, 20].

It's important to note that while our participants' experiences are authentic, they sometimes contained misconceptions. For instance, some women believed stress caused their POI, a belief that is not supported by current scientific evidence. While we've included their quotes to respect their perspectives, we've clarified these points in the discussion to provide readers with accurate, evidence‐based information.

n this study, the concept of “reproductive health needs” was defined as a comprehensive and multifaceted framework that extends beyond a purely clinical approach. This concept encompasses three main dimensions that emerged simultaneously from the participants' experiences: self‐aware needs, which were directly expressed by individuals and included their personal wishes and concerns; unmet clinical needs, which refer to the existing gaps and deficiencies in the provision of medical and therapeutic services; and structural and service gaps, which reflect systemic inefficiencies such as the lack of an accurate registry system or the absence of supportive protocols. This comprehensive approach allowed for a deeper analysis of the challenges faced by women with POI, demonstrating that their reproductive health is influenced by a complex interplay of individual, clinical, and systemic factors.

### 4.6 Strengths and Limitations

The inclusion of both married and unmarried women in this study offers a comprehensive perspective on POI within a culturally sensitive context, where discussing reproductive and sexual needs is often stigmatized. However, cultural barriers may have constrained the willingness of unmarried women to openly discuss their sexual needs. To achieve a more thorough understanding, future research should consider exploring the perspectives of spouses and families, as their viewpoints are essential for a holistic comprehension of this condition. Another limitation of this study is the lack of representation from diverse ethnicities and cultures.

### 4.7 Recommendations

1. The study's findings highlight a critical need for targeted interventions and further research. Based on our analysis, we recommend the following
2. Integrated healthcare programs: Develop and implement integrated care programs for POI within existing health centers to ensure a holistic approach to patient care.
3. Prevalence studies: Conduct large‐scale prevalence studies in Iran to accurately estimate the scale and burden of POI, which is essential for effective public health planning.
4. National registry: Establish a national registry system to systematically identify, track, and support women with POI throughout their reproductive lifespan.
5. Family and community research: Future research should explore the perspectives of spouses, families, and communities to better understand and address the cultural dimensions that impact the lives of women with POI

## 5 Conclusion

In conclusion, this qualitative study highlights the profound and multifaceted challenges faced by women with POI in Iran, which are often intensified by cultural stigma and a lack of accessible, empathetic healthcare. Our findings underscore the critical need for interventions that directly address these lived experiences. Recommendations derived from this research include the establishment of culturally sensitive educational programs for healthcare providers to improve communication and diagnostic processes, as well as the creation of dedicated peer support initiatives to help women navigate the emotional and social impacts of their condition. Ultimately, these findings serve as a crucial foundation for developing patient‐centered care models that prioritize not only the biological aspects of POI but also the psychological and social needs of affected women.

## Author Contributions

Farideh Pargar designed the study, conducted data collection, performed initial coding, analyzed and interpreted data. Zeinab Rabiei drafted the article. Maryam Farjamfar contributed to the study design. Shahrbanoo Goli assisted in data analysis and interpretation. Zahra Motaghi supervised for writing the manuscript.

## Consent

The study was approved by the Ethics Committee of Shahroud University of Medical Sciences (IR. SHMU. FNM. REC.1403.099). Prior to each interview, verbal informed consent was obtained from all participants. They were fully informed about the study's purpose, their right to confidentiality, and their option to pause or stop the audio recording at any time.

## Conflicts of Interest

The authors declare no conflicts of interest.

## Transparency Statement

The lead author Zahra Motaghi affirms that this manuscript is an honest, accurate, and transparent account of the study being reported; that no important aspects of the study have been omitted; and that any discrepancies from the study as planned (and, if relevant, registered) have been explained.

## Data Availability

All data analyzed in this systematic review are derived from previously published studies. All authors have read and approved the final version of the article, had full access to all of the data in this study, and take complete responsibility for the integrity of the data and the accuracy of the data analysis.
